# Combination of the BK-Channel Antagonist ENA-001 and Naloxone Is More Effective Than Either Agent Alone in Reversing Fentanyl-Induced Respiratory Suppression in Rats

**DOI:** 10.7759/cureus.109545

**Published:** 2026-05-24

**Authors:** Monichan Phay, Lia Kwee, Robert B Raffa, Thomas L Miller, Albert Dahan, Joseph V Pergolizzi

**Affiliations:** 1 Neuroscience, Ealden Lab, Philadelphia, USA; 2 Pharmacy, Temple University, Philadelphia, USA; 3 Clinical Development, Enalare Therapeutics Inc., Mullica Hill, USA; 4 Research and Development, Enalare Therapeutics Inc., Mullica Hill, USA; 5 Pain Management, NEMA Research, Naples, USA

**Keywords:** bk channel, carotid body, ena-001, fentanyl, muscle rigidity, naloxone, rat, respiratory depression

## Abstract

Background

Fentanyl overdose in humans produces two life-threatening effects: central μ-opioid receptor (MOR)-mediated hypoventilation and generalized muscle rigidity that further impairs ventilation. Naloxone, the current standard of care, reverses both effects via competitive antagonism at MOR; however, its clinical utility is limited, particularly in the setting of polydrug overdose, where nonopioid mechanism(s) may also contribute to respiratory suppression. ENA-001 restores ventilation as a peripherally directed (carotid body) BK-channel antagonist with a mechanism independent of MOR, and is "agnostic" to the drug cause of respiratory depression. The effectiveness of a combination has not yet been assessed. This study was designed to determine the nature of the pharmacodynamic interaction between naloxone and ENA-001 against fentanyl-induced effects related to overdose toxicity. Specifically, to determine whether their combined effect is neutral, additive, or antagonistic.

Methods

Fentanyl was administered to adult male Sprague Dawley rats as a single intravenous (i.v.) bolus injection via the lateral tail vein following pretreatment with naloxone and/or ENA-001 administered via intramuscular (i.m.) injection. A timer was started at the time of i.v. fentanyl injection, and hypoventilation and muscle rigidity were recorded to define the duration of early ventilatory suppression and the time to recovery from fentanyl-induced body rigidity.

Results

Pretreatment with naloxone and ENA-001 together was more effective (p < 0.001) in attenuating fentanyl-induced respiratory suppression than either agent alone (p < 0.05 and 0.01, respectively), and the combination did not compromise the body rigidity recovery timeline.

Conclusions

In this rat model of fentanyl-induced effects associated with overdose, coadministration of the combination of the opioid antagonist naloxone and the BK channel antagonist ENA-001 represents a potentially more effective treatment of fentanyl overdose than either agent alone.

## Introduction

Illicit fentanyl is now implicated in more than 70% of US opioid-related overdose deaths [1]. Fentanyl is a full μ-opioid receptor (MOR) agonist with approximately 100- to 200-fold greater analgesic potency than morphine and, due to its high lipophilicity (log P = 4.05) and compact molecular weight (336.5 Da), rapidly crosses the blood-brain barrier [2]. These pharmacodynamic (PD) and pharmacokinetic (PK) properties underlie both fentanyl’s clinical utility and its overdose lethality. At high doses, fentanyl produces two mechanistically distinct but lethal effects. First, central MOR activation in the pre-Bötzinger complex, parabrachial nucleus, and nucleus tractus solitarius depresses respiratory rhythm generation, leading to a reduction in respiratory rate, tidal volume, and ultimately minute ventilation to levels incompatible with survival [3,4]. Second, fentanyl produces generalized skeletal muscle rigidity, commonly referred to as the wooden chest syndrome (WCS), through a central mechanism involving the locus cœruleus (LC) and cœrulospinal noradrenergic pathway, which imposes tonically elevated muscle tone on both limb and axial musculature [5,6]. The resulting decrease in chest wall compliance further exacerbates central hypoventilation, amplifies alveolar CO_2_ retention, and impedes reversal [7].

Naloxone, a nonselective MOR antagonist, is the standard pharmacologic intervention for opioid overdose. Naloxone rapidly competes with fentanyl at MOR and can reverse both respiratory depression and muscle rigidity [8]. However, naloxone has several important limitations in the context of high-potency synthetic opioid overdose: its elimination half-life is shorter than that of many illicit fentanyl analogs, creating a risk of renarcotization; reversal of opioid-induced analgesia, precipitation of acute withdrawal; and, critically, little or no benefit against co-intoxicants that depress respiration through nonopioid mechanisms, such as xylazine, benzodiazepines, or propofol [8-10]. These limitations drive active interest in mechanism-agnostic respiratory stimulants.

ENA-001 (formerly GAL-021) is a selective antagonist of large-conductance calcium-activated potassium (big potassium, BK, BK_Ca_, Maxi-K, slo1, or Kca1.1) channels expressed on type-1 glomus cells of the carotid bodies (CBs) [11,12]. Inhibition of these BK channels alters resting membrane potentials to facilitate depolarization of glomus cells, triggering release of excitatory neurotransmitters and increased afferent signaling via the carotid sinus nerve (CSN) to brainstem respiratory centers, thereby mimicking the physiological ventilatory response to hypoxia [13]. Because this mechanism operates independently of the opioid receptor system, ENA-001 reverses respiratory depression regardless of the pharmacological cause and, importantly, does not attenuate opioid analgesia or precipitate opioid withdrawal [14,15].

Combining naloxone and ENA-001 is conceptually attractive: naloxone provides rapid, receptor-targeted reversal of both respiratory depression and muscle rigidity through central MOR antagonism, while ENA-001 provides peripheral, receptor-independent stimulation of respiratory drive. The combination potentially exploits distinct and complementary mechanisms, a prerequisite for PD interaction. Several authors have noted that combining agnostic respiratory stimulants with naloxone represents a promising avenue for rapid overdose reversal, especially in the context of polysubstance co-ingestion [10]. However, no published study has tested whether the naloxone plus ENA-001 combination is neutral, additive, or antagonistic against fentanyl's effects. In the present study, we administered naloxone and ENA-001 alone and in combination to fentanyl-challenged rats and quantified their effects on fentanyl-induced hypoventilation and generalized muscle rigidity. The first systematic documentation of fentanyl-induced posture and cataleptic effects in a rodent model was reported by Malec et al. [16], who demonstrated that fentanyl produced a dose-dependent cataleptic-like state in rats that was fully reversed by the opioid antagonist nalorphine, but was not reversed by the muscarinic acetylcholine receptor antagonist atropine, establishing opioid receptor involvement. In that study, fentanyl's cataleptogenic profile was compared with morphine and codeine and was distinguished by a unique pharmacological signature; whereas haloperidol potentiated morphine- and codeine-induced catalepsy, it moderately augmented fentanyl-induced catalepsy. The catecholamine synthesis inhibitor a-methyl-p-tyrosine also potentiated fentanyl's effects, indicating that catecholaminergic tone tonically suppresses the fentanyl cataleptic state [16]. This early work predated the mechanistic literature on LC involvement and established the behavioral and pharmacological framework that subsequent research elaborated. At the neuroanatomical level, fentanyl-induced muscle rigidity in rats is now understood to be initiated primarily through MOR activation at the LC and propagated through the cœrulospinal noradrenergic pathway to spinal alpha-1 adrenoceptors on spinal anterior horn motor neurons. LC microinjection studies have demonstrated that LC is both necessary and sufficient for fentanyl-induced muscular rigidity, with the underlying ionic mechanism involving a G protein-mediated inhibition of potassium conductance coupled to activation of calcium channels [17,18]. It has been further shown that the cœrulospinal tract carries the descending efferent signal responsible for the increase in skeletal muscle tone [19,20]. This centrally initiated rigidity manifests in the rat as a characteristic postural syndrome: the animal assumes a hunched, truncal-stiff posture with extended limbs, reduced spontaneous movement, and sustained cataleptic immobility when placed on a horizontal bar. The "bar test," first described in the context of neuroleptic-induced catalepsy and subsequently validated for opioid catalepsy, provides a standardized quantitative endpoint [21]. At analgesic doses of fentanyl (10-50 µg/kg i.v. in the rat), catalepsy duration is typically brief (<5 min), reflecting the rapid redistribution of fentanyl from brain tissue. At overdose-relevant doses (150-300 µg/kg i.v.), fentanyl produces persistent tonic muscle contractions affecting the limbs, trunk, and respiratory muscles, a syndrome analogous to the WCS in humans [5,7].

The respiratory consequences of fentanyl-induced muscle rigidity are independent of, and additive to, its central respiratory depression effects. Haouzi and Tubbs [7] measured respiratory system compliance in mechanically ventilated sedated rats receiving overdose-range fentanyl and documented a progressive decrease from baseline at 30 minutes in animals developing persistent rigidity, accompanied by a significant rise in oxygen consumption attributable to the metabolic cost of tonic muscle contraction. The resulting increase in metabolic demand amplifies the hypoxemia beyond that attributable to hypoventilation alone. This dual-pathway toxicity has been proposed to be one explanation for the greater lethality of fentanyl overdose compared to morphine [7]. Naloxone antagonizes both fentanyl-induced muscle rigidity and respiratory depression [8,22], confirming that both effects are MOR-mediated at doses relevant to overdose. The fact that a higher naloxone dose is required to reverse fentanyl-induced rigidity than morphine-induced rigidity in rodents is consistent with fentanyl's distinct MOR and physicochemical properties [22]. The totality of prior observations motivated this study of combination strategy, naloxone plus ENA-001, that, if positive, might reduce the naloxone dose required for rigidity reversal, thereby reducing the risk of precipitated withdrawal while still restoring ventilation.

ENA-001 developed from the structural deconvolution of almitrine, with the difluorobenzhydrylpiperazine moiety removed from the pharmacophore, resulting in a selective, peripherally acting BK-channel antagonist that stimulates breathing without the adverse effects that limit almitrine's clinical use [11,13]. The CBs, the site of ENA-001’s action, are bilateral chemosensory organs located at the bifurcation of the common carotid arteries. They are the principal peripheral O2 and CO2 sensors of the mammalian respiratory control system, contributing 20-30% of the total ventilatory response to hypercapnia and essentially all of the acute ventilatory response to hypoxia [23]. Type-1 glomus cells within the CB express BK channels that serve as a "braking" mechanism on chemosensory activation: under normoxic conditions, these channels maintain glomus cell membrane polarization and limit neurotransmitter release. ENA-001 inhibits this braking mechanism, causing glomus cells to be more sensitive to depolarization, calcium influx, and release of excitatory transmitters (principally ATP and acetylcholine) onto afferent fibers of the CSN. The resulting increase in afferent drive to the nucleus tractus solitarius and other brainstem respiratory groups augments ventilatory response [11,13]. Because the CB pathway is anatomically and functionally distinct from the brainstem MOR-mediated respiratory depression produced by opioids, ENA-001 can stimulate breathing even when central respiratory neurons are pharmacologically activated by fentanyl. This mechanism-agnostic property was demonstrated in preclinical studies in which ENA-001 reversed respiratory depression induced by opioids, propofol, isoflurane, and midazolam in animal models [11,13]. ENA-001 also reversed the respiratory depression produced by a fentanyl-xylazine combination in rats, demonstrating clinically relevant efficacy against the emerging class of polysubstance overdoses that include the veterinary sedative xylazine (an alpha-2 adrenoceptor agonist insensitive to naloxone) [24]. Clinical evaluation of ENA-001 was undertaken by Roozekrans et al. [15] in a randomized, double-blind, crossover proof-of-concept study in 12 healthy male volunteers, in which intravenous (i.v.) ENA-001 dose-dependently reversed alfentanil-induced isohypercapnic respiratory depression, increasing minute ventilation by 6.1 L/min at the low alfentanil/high ENA-001 condition (p < 0.01). Crucially, ENA-001 had no effect on alfentanil-induced sedation or antinociception, a finding with positive implications for clinical use, i.e., unlike naloxone, respiratory rescue is achieved without reversing the analgesic effect of the opioid [15]. A subsequent PK/PD modeling study was published by the same group [14]. Single ascending-dose safety and PK studies in healthy volunteers established that ENA-001’s dose-dependent effect [12]. Most recently, reversal of propofol-induced blunting of the hypoxic ventilatory response by ENA-001 was confirmed in a randomized controlled trial in 14 healthy volunteers by Jansen et al. [25], reinforcing the compound's mechanism-agnostic profile. The totality of this evidence positions ENA-001 as the most clinically advanced peripheral respiratory stimulant available for combination study with naloxone.

## Materials and methods

Animals

Adult male Sprague Dawley rats (~300 grams, 6-8 weeks of age) from Charles River Laboratories (Wilmington, MA) were used for all experiments. Animals were housed in standard laboratory conditions (22 ± 2°C; humidity: ~40-60%) on a 12-hour light/dark cycle with ad libitum access to food (rodent diet: 20, 5053: 20% protein, pelleted, gamma-irradiated maintenance diet formulated for rats and mice), PicoLab (St. Louis, MO), and water. The rats were acclimated for at least three days before drug administration. All procedures were approved by the Mispro Institutional Animal Care and Use Committee (IACUC) and were conducted in accordance with applicable ethical guidelines (IACUC Protocol #: 2024-ENA-01, Amendment #: A-5).

Drugs and dosing

The drugs used were as follows: fentanyl citrate injection, USP (Hikma Pharmaceuticals, NDC 0641-6030-01), stock concentration 50 µg/mL; naloxone hydrochloride injection, USP (Somerset Therapeutics), stock solution 0.4 mg/mL; and ENA-001 (Enalare Therapeutics, Lot: B230309), stock solution 30 mg/mL. If needed, fentanyl and naloxone were diluted with sterile 0.9% saline prior to administration. Animals were ordered to be at the target body weight of ~300 g. Prior to dosing, each animal was weighed and found to be highly consistent with minimal inter-animal variation. Given the uniformity in body weight, dosing volumes were not adjusted per animal

For i.m. administration, rats were manually restrained, and a 30-gauge needle attached to a 1 mL syringe was inserted 5-8 mm into the quadriceps muscle. Injection was performed slowly after confirming the absence of blood return via aspiration. Naloxone alone (3 µg), ENA-001 alone (3 mg), or the combination of both was administered intramuscularly. Fentanyl (10 µg) was injected intravenously within five minutes following i.m. administration. For i.v. administration, the tail was submerged in warm water for approximately two minutes to promote vasodilation. The injection site was cleaned with an alcohol swab, and a 30-gauge needle attached to a 1 mL syringe was inserted into the lateral tail vein. Fentanyl was administered slowly as a bolus, followed by a saline flush. After injection, the needle was withdrawn, and pressure was applied to the injection site using sterile gauze. Animals were then removed from the restrainer and observed immediately for signs of whole-body stiffness and respiratory dysfunction.

A preliminary dose-finding study was conducted to identify the fentanyl dose that consistently induced whole-body stiffness and respiratory suppression. Intravenous doses of 3 µg, 5 µg, and 10 µg were tested. Following each injection, animals were continuously observed to characterize the onset, duration, and resolution of the phenotypic response. Immediately after i.v.. administration, animals showed slow, shallow abdominal-only breathing, consistent with the early hypoventilation phase. The transition to the later hypoventilation phase was marked by the return of chest wall excursions (i.e., restoration of thoracic breathing) while breathing remained shallow and rapid. Whole-body stiffness was characterized by a raised tail and a full-body plank-like posture. Recovery was recorded in a staged sequence: tail relaxation, return of upper body movement, partial lower body movement, and full voluntary locomotion. Full recovery was defined as the return to normal voluntary activity. A timer was started at the time of the i.v. fentanyl injection, and key behavioral transitions were recorded to define the duration of early hypoventilation and the time to full recovery. The 10 µg/rat dose produced the most consistent and prolonged phenotype, allowing for sufficient dynamic range to observe the effects of pharmacological intervention. This dose was selected as the working dose for all subsequent treatment groups and was administered in a fixed volume of 300 µL using a 0.033 µg/µL working stock concentration.

After establishing the fentanyl model, a dose-finding study was conducted to identify the naloxone dose that would result in a 15-30% reduction in recovery time compared to fentanyl alone. Naloxone was administered via intramuscular (i.m.) injection into the left quadriceps at a fixed volume of 100 µL. Doses ranging from 0.1 µg to 2 µg were tested. The 2 µg/rat dose of naloxone consistently reduced the duration of early hypoventilation and time to full recovery relative to fentanyl alone, while remaining within the target reduction range. This dose was selected for use in both the naloxone-only and combination treatment groups.

A dose-finding study was performed for ENA-001 to determine a dose that achieves a 15-30% reduction in recovery time compared to fentanyl alone. ENA-001 was administered via i.m. injection into the right quadriceps at a fixed volume of 100 µL. Several doses were evaluated within the volume limits, not exceeding 200 µL. A 3 mg/rat dose of ENA-001 delivered in 100 µL consistently reduced the duration of early hypoventilation while maintaining acceptable variability across animals. Acceptable variability refers to a dose of ENA-001 that produced a measurable early hypoventilation duration of at least 1.5 min with low inter-animal variability (CV < 15%) and therefore allows the treatment effect to be evaluated in subsequent studies. This dose was selected for use in the ENA-001 only and combination treatment groups. Two parameters were evaluated: duration of early hypoventilation (abdominal-only breathing effort) and time to full recovery (return to normal voluntary locomotion). Within seconds of fentanyl administration, animals showed pronounced whole-body rigidity and entered the early hypoventilation phase, characterized by abdominal-only breathing. This phase lasted between two and four minutes and was followed by a later hypoventilation phase, marked by the reappearance of chest wall movement. Full-body stiffness resolved gradually in a defined sequence: tail relaxation, upper body movement, partial hindlimb movement, and eventual return to normal voluntary locomotion. Whole-body stiffness was considered fully recovered when the animal regained full control of voluntary locomotion. 

Statistical analyses

All statistical analyses were performed using R Statistical Software (R Foundation for Statistical Computing, Vienna, Austria). Prior to hypothesis testing, the normality of each treatment group was assessed using the Shapiro-Wilk test (a = 0.05). Homogeneity of variance was evaluated using Levene’s test (a = 0.05). Since some groups did not meet normality assumptions and/or exhibited unequal variance, nonparametric testing was used. For both primary outcome measures, which are the time to full recovery from fentanyl-induced whole-body stiffness and restoration of thoracic excursion (restoration of thoracic breathing), group differences were assessed using the Kruskal-Wallis test, followed by Dunn’s multiple comparisons test with Bonferroni correction to adjust for multiple pairwise comparisons (Bonferroni-adjusted p-values were used for conservative interpretation of significance). Raw or adjusted p-values < 0.05 were considered statistically significant.

## Results

Fentanyl administration at 10 µg i.v. induced a consistent sequence of behavioral and respiratory changes across all animals. There were no animal deaths. Within seconds of dosing with fentanyl, animals exhibited pronounced whole-body rigidity and entered the early hypoventilation phase, characterized by abdominal-only breathing. This phase typically lasted between two and four minutes and was followed by the later hypoventilation phase, marked by reappearance of chest wall movement. Full-body stiffness resolved gradually in a defined sequence: tail relaxation, upper body movement, partial hindlimb movement, and eventual return to normal voluntary locomotion. Two primary quantitative endpoints were selected to capture these dynamics: duration of early hypoventilation (abdominal-only breathing) and time to full recovery (return to normal voluntary locomotion).

**Figure 1:**
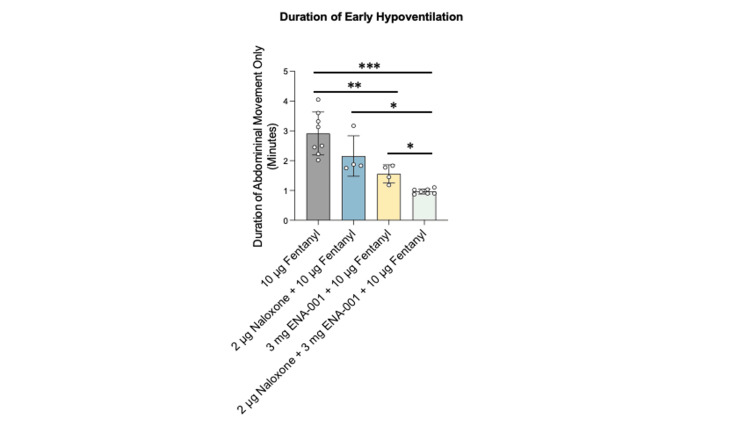
Duration of early hypoventilation following fentanyl administration across treatment groups i.m.: intramuscular; i.v.: intravenous; SD: standard deviation Duration of early hypoventilation following fentanyl administration across treatment groups. Animals received i.m. injection of naloxone (2 μg), ENA-001 (3 mg), or the combination of naloxone (2 μg) + ENA-001 (3 mg), followed by i.v. fentanyl (10 μg). Early hypoventilation was defined as the period of abdominal-only breathing, and its duration was recorded in minutes. Bar plots represent group means ± SD; each dot indicates an individual animal. Statistical analysis was performed using the Kruskal-Wallis test followed by Dunn’s post hoc test. p < 0.05 (*), p < 0.01 (**), p < 0.001 (***)

The duration of early hypoventilation, defined as the period of abdominal-only breathing following fentanyl administration, was significantly affected by pretreatment conditions. Animals that received 10 µg of fentanyl alone showed the longest duration of early hypoventilation. Pretreatment with either naloxone (2 µg, i.m.) or ENA-001 (3 mg, i.m.) reduced this duration relative to fentanyl alone. Coadministration of naloxone (2 µg, i.m.) and ENA-001 (3 mg, i.m.) prior to fentanyl resulted in the shortest duration across all groups. These effects were statistically significant based on Kruskal-Wallis testing followed by Dunn’s post hoc comparison, following assessment of normality and variance using the Shapiro-Wilk and Levene’s tests, respectively. These results suggest that pretreatment with both agents together was more effective in attenuating fentanyl-induced respiratory suppression than either agent alone (Figure 1).

**Figure 2:**
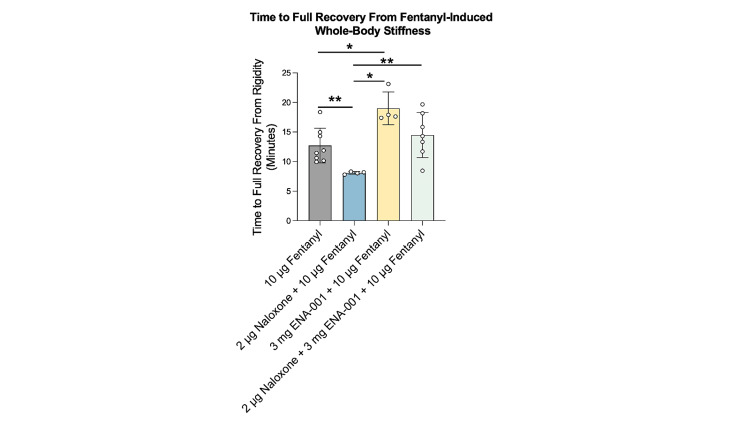
Time to full recovery from fentanyl-induced whole-body stiffness across treatment groups i.m.: intramuscular; i.v.: intravenous; SD: standard deviation Animals received i.m. injection of either naloxone (2 μg), ENA-001 (3 mg), or the combination of naloxone (2 μg) + ENA-001 (3 mg), followed by i.v. fentanyl (10 μg). Time to full recovery from rigidity was measured as the duration from fentanyl-induced whole-body stiffness to the return of normal locomotor activity. Bar plots represent group means ± SD; each dot corresponds to an individual animal. Statistical analysis was performed using the Kruskal-Wallis test followed by Dunn’s post hoc test. p < 0.05 (*), p < 0.01 (**)

Time to full recovery from fentanyl-induced whole-body stiffness was also influenced by pretreatment conditions. Animals pretreated with naloxone (2 µg, i.m.) showed the fastest recovery, significantly shorter than the fentanyl-only group. Coadministration of naloxone (2 µg, i.m.) and ENA-001 (3 mg, i.m.) prior to fentanyl produced a recovery duration comparable to fentanyl alone, but significantly shorter than ENA-001 alone. These group differences were supported by the Kruskal-Wallis analysis followed by Dunn’s post hoc comparison, following confirmation of distribution assumptions. These findings suggest that while naloxone facilitated faster resolution of fentanyl-induced locomotor skeletal muscle rigidity, the combination of both agents did not compromise the recovery timeline (Figure 2).

## Discussion

The present study examined, for the first time, the PD interaction between naloxone and ENA-001 against two manifestations of fentanyl overdose in the rat: hypoventilation and generalized body stiffness. These endpoints were selected because they represent the mechanistically distinct, but clinically important threats that make fentanyl overdose uniquely lethal relative to older opioids, namely, central respiratory depression combined with a mechanical reduction in ventilatory capacity imposed by muscle rigidity [5,7]. The pharmacological rationale for anticipating a beneficial interaction between naloxone and ENA-001 was that naloxone acts at central MOR within the brainstem respiratory network and, via LC-mediated pathways, reverses the cœrulospinal activation that drives muscle rigidity [8,19,20], whereas ENA-001 acts peripherally at carotid body BK-channels to restore peripheral afferent drive to brainstem respiratory controllers independently of MOR occupancy [11,15]. These diverse sites of action do not overlap, fulfilling one of the key theoretical conditions for synergy: distinct receptor mechanisms converging on a shared effect [26].

From a clinical translation standpoint, PD synergy between naloxone and ENA-001 would carry significant practical implications. Of note, Dahan and colleagues have explicitly identified the combination of agnostic respiratory stimulants with naloxone as a priority area for synergy testing, noting that mechanistic complementarity (central MOR antagonism combined with peripheral CB stimulation) is precisely the type of pharmacological configuration most likely to generate synergy rather than simple additivity [10]. If the combination ED50 for each agent is substantially below its individual ED50, it may be possible to achieve complete overdose reversal with a naloxone dose that does not precipitate acute withdrawal syndrome in opioid-dependent patients, which is a major limitation of current naloxone protocols, particularly given the large or numerous naloxone doses required to reverse potent synthetic opioids such as carfentanil and other fentanyl analogs [8,9]. Simultaneously, ENA-001 would provide coverage against any co-intoxicant (e.g., xylazine, benzodiazepine, propofol) that contributes to respiratory depression through non-opioid pathways, addressing a critical gap in current overdose management, particularly polydrug overdose [10,24]. An important consideration would be whether synergy is or is not observed equivalently for both the ventilatory and body stiffness endpoints. These endpoints reflect partially overlapping, but not identical, neuroanatomical circuits: minute ventilation integrates respiratory rhythm generation (e.g., pre-Bötzinger complex, parabrachial-Kölliker-Fuse nuclei, etc.) and chemosensory drive that involves central and CB chemoreceptors, whereas body stiffness is determined mainly by the LC-cœrulospinal-alpha-1 adrenoceptor axis [3,4,18,19]. ENA-001's selective CB mechanism would be expected to contribute more directly to the ventilatory endpoint; its contribution to rigidity reversal would be indirect, operating through the improved arterial oxygenation that secondarily reduces reflex excitation of motor circuits. This mechanistic asymmetry may produce different interaction profiles for the two endpoints, a finding of considerable theoretical and translational interest if confirmed.

Several limitations of the present study should be acknowledged. First, the rat model and small sample size. Although the rat model is the most extensively validated preclinical model for fentanyl-induced catalepsy and respiratory depression, it may not fully recapitulate the dose-response relationships or receptor expression patterns of humans, particularly with respect to the degree of CB contribution to respiratory drive [23]. Second, the fixed-ratio design characterizes the interaction at one dose ratio; additional fixed-ratio doses would be required to map the full interaction landscape and identify the dose ratio(s) producing potential synergy, as recommended by Tallarida [27,28]. The term "synergy" is used widely in the pharmacologic literature, but its rigorous definition requires a quantitative null hypothesis, a prediction of the effect that the combination of two drugs should produce if they interact solely according to their individual effects, without positive or negative interaction. A rigorous mathematical and widely adopted framework for this purpose is Loewe additivity, implemented graphically by isobolographic analysis, as expanded and systematized by Tallarida and colleagues [26,27,29]. An isobologram is constructed by plotting on a Cartesian plane all pairs of doses (a, b) of drugs A and B that, in combination, produce a specified level of effect (typically the effect produced by the ED50 of each drug alone). The intercepts of the Cartesian axes are the ED50 values for each drug used alone. Under the null hypothesis of simple additivity, the predicted additive isobole is the straight line connecting the two intercepts. Experimental combination dose pairs that fall below this line (i.e., a smaller total dose than predicted by additivity achieves the target effect) indicate synergy; pairs above the line indicate subadditivity or antagonism [26,29]. When the individual dose-effect curves have a constant potency ratio, they are parallel on a log-dose scale, and the additive isobole is linear. When the potency ratio varies (e.g., when one agent is a full agonist and the other a partial agonist, or when the two drugs act at mechanistically distinct systems with non-parallel dose-response curves), the additive isobole may be curved, and its construction requires the methods of Grabovsky and Tallarida [29]. For the present study, in which naloxone and ENA-001 both shift the dose-effect curve of fentanyl's respiratory and postural effects, but do so through distinct systems, nonparallel dose-effect curves and a varying potency ratio are plausible. Hence, ED50 determination for each drug alone and for a fixed-ratio combination is required [26,27]. The fixed-ratio design, in which the two drugs are coadministered at a constant dose ratio determined a priori from their individual ED50 values, is standard practice because it permits the generation of a single combination dose-effect curve, allowing direct calculation of ED50, mix, and its confidence interval for comparison with the additive prediction [27,29]. Statistical significance is assessed by testing whether the 95% confidence interval of ED50, mix falls below the lower confidence boundary of the additive isobole at the tested dose ratio, using the difference between observed and predicted additive total doses [27,29]. This framework has been applied across a diverse range of pharmacological contexts, including opioid combinations, analgesic mixtures, anti-infective regimens, and combination overdose reversal strategies [28,30]. Future study should test this hypothesis directly, applying the isobolographic framework to both ventilatory and postural endpoints.

Other limitations include that potential PK interactions between naloxone and ENA-001 were not assessed; PK-mediated changes in tissue drug concentrations could, in principle, contribute to apparent PD interaction and should be addressed in future mechanistic studies. Also, the single-dose design limits interpretation, and the absence of full dose-response relationships weakens translational relevance. Reliance on qualitative respiratory endpoints without objective measurements (e.g., blood gases, ventilation metrics) reduces scientific rigor; no power analysis was performed. The rat model's translational applicability is insufficiently justified, particularly regarding carotid body differences. Lack of blinding, randomization, and PK interaction assessment introduces bias, and the re-treatment design limits clinical relevance.

Despite the limitations, this study using this rat model of fentanyl-induced effects associated with overdose, coadministration of the combination of the opioid antagonist naloxone and the BK channel antagonist ENA-001 represents a potentially more effective treatment of fentanyl overdose than does either agent used alone.

## Conclusions

In this rat model of fentanyl-induced respiratory and motor suppression, combined administration of naloxone and ENA-001 prior to fentanyl challenge produced the greatest inhibition of early hypoventilation duration and whole-body rigidity recovery profile comparable to naloxone alone. While each agent individually mitigated aspects of fentanyl-induced effects, the combined treatment more effectively reduced the duration of respiratory suppression while preserving the recovery from locomotor skeletal muscle rigidity.
